# LMN Facial Palsy in Pregnancy: An Opportunity to Predict Preeclampsia—Report and Review

**DOI:** 10.1155/2014/626871

**Published:** 2014-03-17

**Authors:** Vani Aditya

**Affiliations:** Type-4/13, BRD Medical College, Gorakhpur, Uttar Pradesh 273013, India

## Abstract

Facial paralysis is the most frequent unilateral cranial nerve pathology affecting pregnant population 2 to 4 times more often than the nonpregnant population. There exists an association with preeclampsia but this has largely been overlooked. Clinicians often dismiss it for idiopathic palsy as seen in the present case. A 30-year-old woman, Gravida 4, Para 3, presented at 26 weeks pregnancy with complaints of facial weakness, blurring of vision, altered taste sensation, increased noise sensitivity for 1 month, headache since 18 days, and vomiting since 2­3 days. Her pulse was 90/min, BP was 170/120, and RR was 18/min. Uterus was 18 weeks size and proteinuria++ was present. Ultrasonography revealed a 26 weeks fetus, severe bradycardia, and absent liquor. HELLP syndrome was diagnosed after investigations. Six units of fresh frozen plasma were transfused. An informed decision for termination of pregnancy was made. She delivered a 450 gram stillborn. The third stage was complicated with postpartum hemorrhage but it was managed successfully. Women with Bell's palsy during pregnancy should be evaluated critically as in some it may precede preeclampsia which has serious maternal and fetal implications. Therefore, these women should be in regular followup of the obstetrician.

## 1. Introduction

Facial paralysis is an entity that most neurologists and otolaryngologists are familiar with. It is the most common and frequent unilateral cranial nerve pathology. Most commonly it is idiopathic or Bell's palsy named after Sir Charles Bell who first described this condition and also its association with pregnancy [1].

Bell's palsy is also associated with preeclampsia but this has been overlooked in the past. The patients have not been followed by the neurologists for such an event. The obstetricians, who often recognize central facial paralysis in severe preeclampsia as a part of stroke, have failed to relate peripheral facial palsy to preeclampsia only to dismiss it for idiopathic palsy as seen in the present case.

## 2. Case

A 30-year-old, gravida 4, para 3, woman presented in antenatal outpatient department (OPD) at 26 weeks pregnancy with complaints of facial weakness, blurring of vision, altered taste sensation, and increased noise sensitivity for 1 month. She had headache since 18 days and vomiting since 2-3 days. All her previous deliveries were at home. In her last delivery at term, she had a stillborn baby.

The present case was diagnosed with hypertension when she was three months pregnant and took prescribed antihypertensive drugs for about two months. About twenty days after stopping medication she developed facial weakness which progressed over the following 2 to 3 days. There was no history of fever, rash, trauma, or symptoms of dental/ear infection. Before visiting our institution she visited two private practitioners. Both advised admission in view of intrauterine growth restriction and oligohydramnios though her blood pressure records were normal. Finally, she visited a primary health centre from where she was referred to our tertiary care centre.

On examination the patient was conscious but kept her eye and ears covered. Her pulse was 90/min, BP was 170/120, RR was 18/min, and chest and cardiovascular examination was normal. Uterus was 18 weeks size, fetal heart rate was 100/min, and ++proteinuria was present. Opinion was taken from the neurologist regarding Bell's palsy. She was advised physiotherapy and prescribed with multivitamins.

The patient was prognosticated about the high risk condition and poor fetal outcome. She consented to admission and treatment. Antihypertensive (labetalol) and anticonvulsant (magnesium sulphate) were started. Ultrasonography revealed a single live fetus consistent with 26 weeks gestational age, with absent liquor and severe bradycardia. Baseline investigations revealed SGOT 248, SGPT 235, platelet count 77,000/*μ*L, INR 1.5, and aPTT ratio 1.5. Diagnosis of HELLP (hemolysis, elevated liver enzymes, and low platelets) syndrome was made.

An informed decision was made to terminate the pregnancy. Six units of fresh frozen plasma (FFP) were transfused. Once the coagulation profile became normal, cervical ripening and labor induction were commenced. After 6 hours, she delivered a 450 gram stillborn. The third stage was complicated with postpartum hemorrhage with a blood loss of about 1500 to 2000 mL. The hemorrhage was successfully managed and four more units of FFP and 2 units of packed cell volume were transfused. The postnatal period was uneventful. She was evaluated for hypertension. On ultrasonography, the left kidney was found to be atrophied. Urinary protein over 24 hours was 546 mg. Fundoscopy showed pale discs.

On follow-up visit after 47 days, her palsy was noted to improve partially though her blood pressure remained elevated (220/120). ECG showed a left axis deviation. Thereafter she was advised regular followup in departments of general medicine and neurology.

## 3. Discussion

The incidence of Bell's palsy is 17/100,000 population per year in women of child bearing age (WCBA). The pregnant population is affected about 2 to 4 times more than the nonpregnant population with an incidence of 38 to 45/100,000 deliveries [2]. However, in a given year, in a population of 100,000 WCBA there will be 6400 births and therefore 17 cases of Bell's palsy in WCBA but only 3 cases of Bell's palsy in the pregnant population [3].

**Figure 1:**
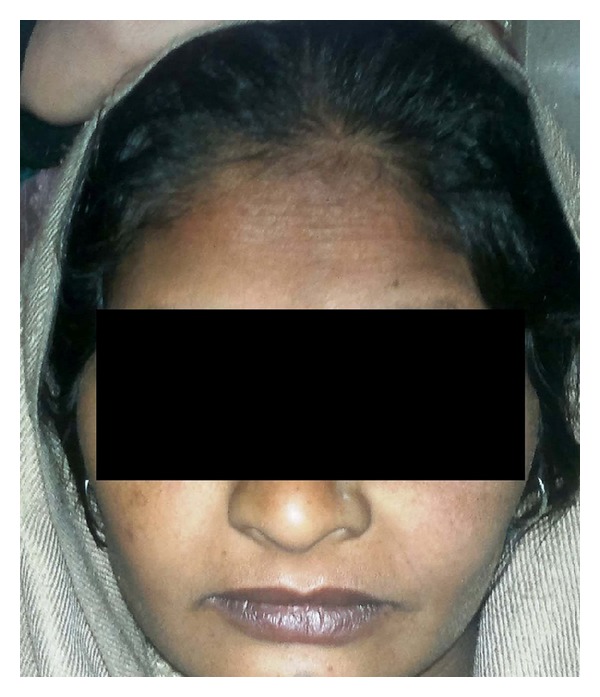
Flat nasolabial fold and inability to wrinkle the forehead (right side).

**Figure 2:**
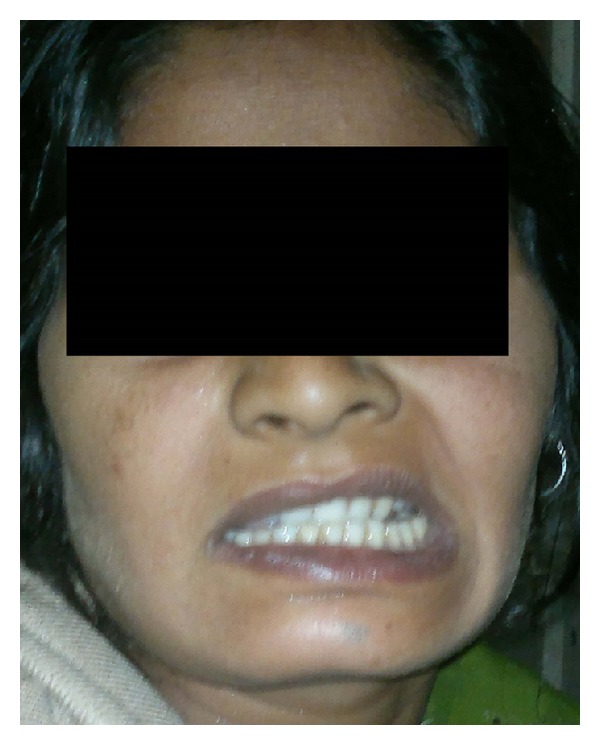
Inability to show teeth (right side).

**Figure 3:**
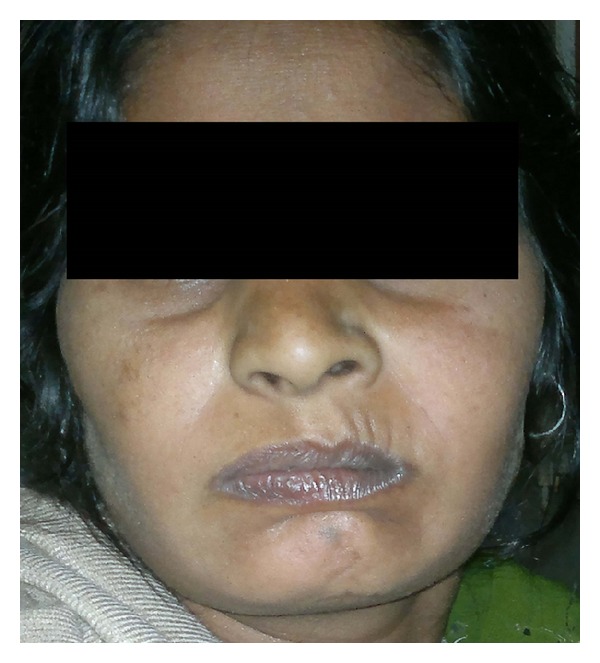
Inability to purse lips (right side).

**Figure 4:**
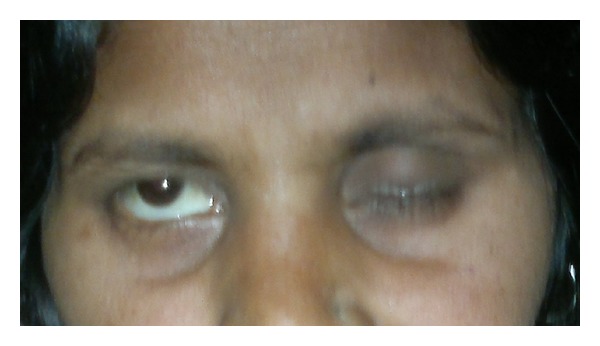
Bell's phenomenon (upward rolling of eye ball).

Clinical presentation of Bell's palsy in pregnancy is the same as in nonpregnant state. The typical findings are less prominent wrinkles on the affected side, eyebrow droop, flattened nasolabial folds, corner of the mouth turned down and inability to wrinkle forehead, raise eyebrows, purse lips, show teeth, or whistle (Figures 1, 2, and 3). Eye closure may be incomplete, and on attempting to close the lid there is upward displacement of the eyeball exposing part of the sclera: “Bell's sign” (Figure 4). There may be dryness of eyes, changes in taste in anterior 2/3rd of tongue, noise sensitivity, and difficulty in salivation.

About 15% of pregnant patients with acute facial paralysis may have etiologies other than Bell's palsy [4]. Thorough history and examination are paramount to narrow the differential diagnosis. Often Bell's palsy is mistaken for stroke because of its sudden onset, and because it results in numbness and loss of muscular control on the affected side. However, in upper motor neuron (UMN) lesions, for example, cardiovascular accident like stroke, upper third of the face is spared while in lower motor neuron (LMN) lesions, for example, Bell's palsy, there is paralysis of the entire face.

A large number of studies have observed the association of Bell's palsy with pregnancy. Most cases are confined to the third trimester and the immediate postpartum period [5]. Several physiological changes during pregnancy have been proposed to explain this association.

Pregnancy is maximally immunocompromised in the last trimester from rising titers of cortisol. Herpes virus, which lives in the geniculate nucleus of the facial nerve, gets activated in conditions of compromised immunity. This sets in an inflammatory reaction that directly damages nerves by demyelination. As majority of cases of Bell's palsy are observed during third trimester, viral infection may be a plausible cause [6]. Sometimes vesicles may not appear in HZ infection and herpes is now considered to be the cause of facial palsies in one third of cases previously diagnosed as idiopathic [7]. Reactivation of oral HSV is also seen with the use of epidural or intrathecal morphine. Thus some cases of postpartum Bell's palsy may be a consequence of anesthetic management [8].

An increase in the interstitial fluid and peripheral edema in pregnancy results from plasma volume expansion and venous stasis. This leads to compression neuropathies in places where the nerves course in a closed space like carpal tunnel syndrome [6]. In the fallopian canal, tissue edema causes mechanical compression of the facial nerve. This hypothesis is supported by the peak incidence of Bell's palsy coinciding with the gestational age at which there is maximal interstitial fluid, that is, in third trimester [9, 10]. In the postpartum period, the plasma volume returns to normal faster than the interstitial fluid volume which leads to venous congestion and edema in the narrow fallopian canal.

Increase in clotting factors in pregnancy results in a hypercoagulable state and thrombosis of vasa nervosum supplying the facial nerve can cause devascularization and ischemic nerve injury [8]. Estrogen and progesterone effects have also been proposed to cause Bell's palsy in pregnancy [11]. Some workers have argued a familial tendency for idiopathic facial nerve pathology [10, 12].

Bell's palsy during pregnancy has been associated with preeclampsia [5, 13]. Physiological changes involved in pathogenesis of Bell's palsy during pregnancy are common with the etiopathogenesis of preeclampsia which explains the high incidence of preeclampsia in women with Bell's palsy. Preeclampsia often manifests with considerable edema and many women may have underlying thrombophilia, which exaggerates the hypercoagulable state of pregnancy. Importantly, in a patient with preeclampsia, facial palsy may be peripheral or central, from stroke, and the two should be differentiated because their management differs greatly.

Though it is not clear whether Bell's palsy is temporally related to preeclampsia, in many cases [5, 14–17] Bell's palsy is seen to occur before the onset of severe preeclampsia, superimposed preeclampsia, or HELLP (Table 1). It is possible that the edematous state in pregnancy, which is exaggerated in preeclampsia, manifests as isolated neurological deficit among which Bell's palsy is the most obvious one. Other features of preeclampsia like hypertension and proteinuria may manifest later. Bell's palsy has also been reported to occur after the onset of preeclampsia though in all the reported cases [12, 16, 18] facial palsy occurred in the postpartum (Table 2). Some women with preeclampsia may have underlying thrombophilia and in the postpartum period when the hypercoagulability is also maximum; from physiological increase in clotting factors, their risk of thrombosis is highly increased. Thrombosis of vasa nervosa of facial nerve leads to Bell's palsy.

In our case, there was history of hypertension and intake of hypertensive drugs in the first trimester but the reason why proteinuria and severe oligohydramnios were not correlated with the hypertensive disorder during two of her previous OPD visits in the second trimester was normal blood pressure record even without the intake of antihypertensive drugs. The facial palsy was presumed to be Bell's palsy. Overall, an opportunity to prevent the impending doom was missed and diagnosis of preeclampsia superimposed on chronic hypertension was suspected only after there was a rise in blood pressure in the late second trimester. The blood pressure records were normal in previous OPD visits due to the physiological mid-trimester fall.

Treatment of Bell's palsy includes topical eye care, corticosteroids, and/or antiviral with surgery being remotely needed. Use of steroids has been found to improve recovery [19, 20]. Some authors consider treatment with steroids as superfluous [5, 21]. High rate of spontaneous recovery after delivery has been observed and this has been associated with higher levels of endogenous steroids during pregnancy, younger age of this population, and resolution of physiological and anatomical changes after birth of the baby. Yet delivery by elective induction to improve outcome of Bell's palsy or prevent onset of preeclampsia is not indicated. This can result in prematurity and increased chances of caesarean birth. Elective induction should remain reserved for obstetric indications only. Whether drugs like magnesium sulfate used in preeclampsia can actually worsen the recovery of Bell's palsy needs to be investigated [22].

Women with Bell's palsy during pregnancy should be evaluated critically as in some it may precede preeclampsia which has serious maternal and fetal implications. They should also be regularly followed up by obstetrician.

## Figures and Tables

**Table 1:** Antepartum Bell's palsy.

Author	Cases (*n*)	Age (years)	GA at onset of Bell's palsy (weeks)	Hypertensive disorders	Time interval from onset of Bell's palsy to diagnosis of preeclampsia (days)
Shapiro et al. (1999) [5]	2	23	32	Preeclampsia	2
		35	32	Preeclampsia	14
				HELLP	
Juan et al. (2010) [14]	1	38	37	Superimposed preeclampsia	10
Cabrera et al. (2012) [15]	1	29	36	Preeclampsia	<1
Pourrat et al.(2013) [16]	2	35	31	Preeclampsia	14
		27	34	Preeclampsia	14
Ragupathy and Emovon (2013) [17]	2	40	39	Preeclampsia	4
		26	37	Preeclampsia	NA
Present case	1	30	22	Superimposed preeclampsia + HELLP	30

**Table 2:** Postpartum Bell's palsy.

Author	Cases (*n*)	Age (years)	GA at onset of Bell's palsy (weeks)	Hypertensive disorders	Time interval from onset of Bell's palsy to diagnosis of preeclampsia (days)
Mathieu and Ledigabel (2011) [18]	1	22	40	HELLP	1
Mylonas et al. (2005) [12]	1	35	30	Preeclampsia	7
Pourrat et al. (2013) [16]	1	29	39	HELLP	4
